# Dynamic alterations and clinical implications of the plasma proteome in pediatric sepsis

**DOI:** 10.1186/s40001-025-02933-5

**Published:** 2025-07-23

**Authors:** Shiyuan Fan, Xinglv Liu, Zichi Zhao, Yanjuan Liu, Yu Jiang, Saizhen Zeng

**Affiliations:** 1 Hunan Provincial People’s Hospital and The First Affiliated Hospital of Hunan Normal University, 61 Jie-Fang West Road, Fu-Rong District, Changsha, 410005 Hunan People’s Republic of China; 2 Hunan Provincial Hospital of Integrated Traditional Chinese and Western Medicine (Affiliated Hospital of Hunan Academy of Chinese Medicine), Changsha, 410006 China

**Keywords:** Children, Sepsis, Plasma, Mass spectrometry, Proteome

## Abstract

**Background:**

Current sepsis biomarkers have limitations, but mass spectrometry-based proteomics can identify patients at high risk of mortality or organ dysfunction, identify the molecular mechanisms of pediatric sepsis, and reveal personalized biomarkers and therapeutic strategies, with high-risk cohorts benefiting from early and accurate identification through clinical biomarkers.

**Methods:**

The young mice were randomly divided into sepsis and sham groups(D0), and then the plasma was dissected at D0, Day 1(D1), Day 3(D3), and Day 7(D7) after surgery for additional protein identification by liquid chromatography-mass spectrometry (LC/MS) proteomics. Subsequently, data from 66 cases of children diagnosed with sepsis upon admission to Pediatric Intensive Care Unit at Hunan Provincial People's Hospital and The First Affiliated Hospital of Hunan Normal University were gathered. Dynamic plasma samples (D1, D3, D7) were obtained for ELISA verification and correlation analysis of the candidate biomarkers to determine the clinical significance of sepsis candidate plasma biomarkers.

**Results:**

Among the 6578 proteins identified, the septic mice groups (D1, D3, D7) demonstrated 161 differently upregulated plasma proteins. The main enriched pathways in the KEGG study were related to complement and coagulation cascades, focal adhesion, and phagosomes. ELISA test results indicated that among pediatric patients, the five candidate biomarkers (AT III, CFD, Col1α1, EGFR, Thbs1) all showed varying degrees of decrease in diagnosing sepsis. Correlation study results suggested that AT III was adversely linked with IgA, IgG, IgM, C3, with Pearson's coefficients of −0.543, −0.217, −0.526, −0.128, respectively. CFD was positively connected with IgA, IgG, IgM, and negatively correlated with C3. Col1α1, CFD, EGFR, and Thbs1 demonstrated negative correlation with suppressive CD8 + cells, while Col1α1, EGFR, and Thbs1 showed positive correlation with B cells (CD19 +). Furthermore, Col1α1, CFD, EGFR, and Thbs1 revealed positive connection with CD4 + /CD8 + . Additionally, AT III demonstrated positive connection with PT, APTT, INR, D-Dimer, and Fbg. Conversely, Col1α1 and EGFR showed negative association with PT, APTT, INR, D-Dimer, and Fbg. CFD was positively correlated with Fbg, and Thbs1 showed positive correlation with D-Dimer.

**Conclusion:**

Within 1 week of sepsis onset, 161 proteins revealed alterations in young mice, with the complement and coagulation cascades, focal adhesion, and phagosome pathways showing the most significant correlations. All prospective markers reduced following the recognition of sepsis and were associated with coagulation and immunological function in pediatric patients.

**Supplementary Information:**

The online version contains supplementary material available at 10.1186/s40001-025-02933-5.

## Introduction

Sepsis, defined as life-threatening organ failure induced by an aberrant host response to infection, remains a substantial clinical challenge [1]. The criteria for pediatric sepsis proposed by the International Pediatric Sepsis Consensus Conference in 2005 have been widely used in clinical practices, research, quality improvement programs, and policy frameworks [2]. In January 2024, the Pediatric Sepsis Definition Task Force of the Society of Critical Care Medicine developed the most recent international consensus guidelines for pediatric sepsis and septic shock [3]. Sepsis remains a common cause of pediatric mortality and pediatric intensive care unit (PICU) admissions [4]. Sepsis is expected to have impacted 48.9 million individuals worldwide in 2017, with 20.3 million cases comprising children under the age of five [5]. As a result, sensitive early clinical indicators are critical for aiding early screening, identification, and intervention in high-risk people. Early and exact identification of at-risk populations is crucial for maximizing treatments among those who will benefit most from prompt therapeutic measures [6].

In the early stages of hospitalization, distinguishing between sepsis and sterile inflammation could be challenging. Furthermore, the microbiological identification of pathogens does not necessarily mean that they are the cause of sepsis, because microbiological laboratories cannot distinguish between colonizing organisms and infectious pathogens [7]. Given the complexity of infection and host–pathogen interactions, a single biomarker is unlikely to yield sufficient precision for infection diagnosis [8]. In addition, host–pathogen interactions during the microbial cell life cycle, including cell entry, pathogen replication, and dissemination, as well as host defense mechanisms, involve certain changes in cell metabolism, which can be detected through key proteins and metabolites [9]. In other words, these novel mass spectrometry (MS) technologies make it possible to obtain a molecular image of infection from both the host and pathogen perspectives. Meanwhile, proteomics can analyze multiple proteins simultaneously, thus generating diagnostic and prognostic proteomic signatures. The future prospect of proteomics lies in its application in precision medicine.

Plasma proteomics research in pediatric sepsis is still in its early phases, with no studies showing the dynamic changes in plasma proteins. Plasma proteome profiling in sepsis has demonstrated that the changes in protein abundance found in a mouse sepsis model largely correspond with the trends documented in human sepsis literature [10]. Consequently, the strong proteomic signals derived from the murine sepsis model are expected to enhance and advance future human-targeted proteome research. In order to screen and uncover possible plasma biomarkers for pediatric sepsis, we performed proteome investigations of plasma protein expression profiles in young sepsis murine models using liquid chromatography-mass spectrometry (LC/MS)-based techniques. Concurrently, we collected data on pediatric sepsis patients initially identified and treated in the PICU of Hunan Provincial People's Hospital and The First Affiliated Hospital of Hunan Normal University. Fundamental admission details and laboratory findings were recorded, and dynamic plasma samples (D1, D3, and D7) were collected. Consequently, Enzyme-Linked Immunosorbent Assay (ELISA) was employed to investigate potential plasma biomarkers of sepsis, aiming to identify distinctive plasma biomarkers for pediatric sepsis and assess their clinical significance within this setting.

## Materials and methods

### Design of experiments

Two distinct experiments were conducted. Experiment 1 established a gold-standard animal model of pediatric sepsis via the cecal ligation and puncture (CLP). Proteomic techniques were employed to identify potential plasma biomarkers for sepsis. In Experiment 2, the potential biomarkers were validated in plasma samples from pediatric patients with sepsis via ELISA. Proteomic analysis, ELISA validation, and clinical evaluation were conducted in a blinded way.

### Animals experiment

Male C57BL/6J mice were procured from Hunan Saike Jingda Experimental Animal Co., Ltd., aged 3–4 weeks, with a body weight of 11.4 ± 0.9 g. In reference to prior studies [11] on the 7-day survival rate in CLP-induced sepsis, the sample size for the sepsis group was set at 40 animals per group. The animals were randomly apportioned into a sham operation group (D0, *n* = 20) and a sepsis group (CLP), further subdivided into Day 1 (D1, *n* = 40), Day 3 (D3, *n* = 40), and Day 7 (D7, *n* = 40) groups. Before the experiment, all animals were acclimatized for a week in SPF animal facilities under controlled conditions of 25–26 °C and a 12:12-h light–dark cycle. Water and food were provided ad libitum. Mice in the CLP subgroups were euthanized on D1, D3, and D7 post-CLP, whereas the D0 group was euthanized 24 h after the sham procedure.

CLP has emerged as the predominant model for experimental sepsis and is considered the gold standard in sepsis research [11, 12]. Mice were anesthetized using 2–3% isoflurane (Ringpu Bio, China). The cecum was exposed utilizing a midline surgical incision and ligated by a 4.0 silk suture. Then, a 21G needle penetrated the cecum and the abdomen was closed in layers with 4.0 sutures. The cecum was mobilized without the employment of CLP for the sham-operated animals. The animals were resuscitated by subcutaneous injection of a prewarmed normal saline (37 °C, 50 mL/kg) at the end of the surgical procedures; then mice were returned to cages immediately where accessed to water and food in freedom, with a temperature-controlled environment (22 °C) for 12 h light and dark cycles. The plasma was obtained post-anesthesia, and blood was extracted from the enucleated eye with an anticoagulant-treated tube to collect roughly 1 ml of blood (*n* = 11/12 per group). Subsequently, blood was centrifuged at 4 °C for 15 min at 3000 rpm and stored at −80 °C until required.

### Proteomic analysis

After equilibrating the samples to room temperature, 10 µl of plasma from each sample was taken and subjected to high-abundance protein depletion using a protein centrifuge column pool. The plasma samples were then diluted with 8 M urea for disulfide reduction and alkylation. Proteins were digested with trypsin (ThermoScientific, US) at 37 °C overnight, followed by peptide purification and desalting. The desalted peptides were dried under vacuum and reconstituted in mass spectrometry (MS) buffer. The samples were subjected to chromatographic separation and analyzed by an Orbitrap Exploris 240 (ThermoScientific, US) MS. The analysis was conducted over 150 min in positive ion mode, with a precursor ion scan range of 350–1200 m/z and a MS1 resolution of 60,000. The AGC target was set to Standard. Peptide and fragment m/z ratios were acquired using the following parameters: Data-independent acquisition (DIA) was set to Top N, with N set to 30. MS2 activation was performed using HCD, with an isolation window of 1.6 m/z. The MS2 resolution was set to 15,000, microscans to 1, and the ion dynamic exclusion time was set to Auto, with a normalized collision energy of 30%.

This study employed a label-free quantitative proteomics approach using DIA-MS1 data integration, a methodology that does not rely on labeled reagents. The DIA-NN software (version 1.8.1) was used to intelligently identify peptide characteristics from the raw data. Following data collection, by setting the grouping, database, and post-translational modification types, DIA-NN computed peak integration intensities to provide label-free quantification data. Additionally, the software filtered the data based on the false discovery rate (FDR) principle, automatically matched the data to the database, and thus yielded highly reliable qualitative results. Finally, the UniProt database was employed to consolidate all quantitative and qualitative results, completing the comprehensive proteomic data analysis.

### Bioinformatics analysis

In the MS assay, each sample underwent three replicates of global protein quantification, yielding three quantitative values. The final quantitative value for each sample was determined as the mean of these three replicates. The ratio of the final quantitative values between different samples was then calculated as the differential expression level Fold Change (FC) for the comparison groups. Analysis and volcano plot generation were carried out using relevant R packages in R version 4.3.1. The log2 (FC) threshold of > 0.5 or < −0.5, combined with *P* < 0.05, was set to determine the upregulated or downregulated protein expression changes in the plasma.

Enrichment analysis for Gene Ontology (GO) annotations and Kyoto Encyclopedia of Genes and Genomes (KEGG) pathways was conducted using the pathview and clusterProfiler R packages provided by the microbioinformatics platform for pathway-based data integration and visualization (https://www.bioinformatics.com.cn). Protein–protein interaction (PPI) data for differentially expressed genes were obtained from the online STRING database (http://string-db.org). The MetaboAnalyst 6.0 platform (https://www.metaboanalyst.ca/MetaboAnalyst/) was used for biomarker feature analysis and pattern prediction.

### Patient recruitment

This study recruited pediatric patients diagnosed with sepsis in the PICU of Hunan Provincial People’s Hospital and The First Affiliated Hospital of Hunan Normal University between January 1, 2023 and December 31, 2023. The selection was based on the definitions of sepsis and related pediatric organ dysfunction standards established at the 2005 International Pediatric Sepsis Consensus Conference. The inclusion criteria consisted of children aged between 28 days and 18 years who were diagnosed with sepsis. The exclusion criteria included the following: (1) children with genetic metabolic disorders or chromosomal diseases; (2) children with primary immunodeficiencies; (3) children who had been on long-term corticosteroids or immunosuppressants; (4) children with malignant tumors; (5) children who were transferred to other medical institutions for various reasons during hospitalization; and (6) children with incomplete clinical data.

Plasma samples were collected and preserved as follows: On D1, D3, and D7 of hospitalization, 2 mL of blood was drawn from each patient into EDTA purple cap tubes in the morning. The collected samples were allowed to sit for 30 min before being centrifuged (3000rpm, 10 min) within 2 h. The supernatant was then stored at −80 °C for further analysis.

### ELISA validation

The plasma samples, after being retrieved from the −80 ℃ freezer, is thawed in a 4 ℃ refrigerator. The blood and intratumour levels of antithrombin III (AT III, encoded by the serpinc1 gene), complement factor D(CFD), collagen type I alpha 1 chain (Col1α1), epidermal growth factor receptor (EGFR), and thrombospondin 1 (Thbs1) were detected with ELISA kits (MultiSciences) following the manufacturer’s instructions.

### Statistical analysis

Bioinformatics and statistical analyses of the proteomics results were performed using the Perseus statistical suite (version 2.0.10.0) within the MaxQuant computational platform. After importing the mass spectrometry identification results into Perseus, filtering was conducted based on peptide counts (> 2) and MS/MS scan counts (> 2) for each peptide. Missing value imputation was done using a group-specific valid value proportion threshold (requiring at least one sample group D0 + D1, D0 + D3, or D0 + D7) to meet the 70% validity criterion), with residual missing values imputed via normal distribution simulation (width parameter 0.3, down-shift 1.8). PBS-perfused samples served as controls for background noise subtraction, and only proteins with an enrichment fold change ≥ 2 in the labeled samples were retained. Two-way ANOVA assessed inter-group significance, with multiple-testing correction via permutation-based FDR (FDR < 0.05, truncated after 250 random permutations). FDR correction, through the Benjamini–Hochberg algorithm, transformed Type I errors from family-wise error rate (FWER) to FDR, enhancing interpretability in high-dimensional data.

Statistical analysis and data visualization were conducted using software GraphPad Prism 9.0 and SPSS 25.0. Data are presented as mean ± SD. Differences between two groups were assessed using Student's *t*-test, while differences among multiple groups were analyzed using one-way ANOVA. *P* < 0.05 was considered statistically significant, and *P* < 0.01 was considered highly statistically significant.

## Results

### Differentially expressed proteins (DEPs) in sepsis plasma

**Fig. 1:**
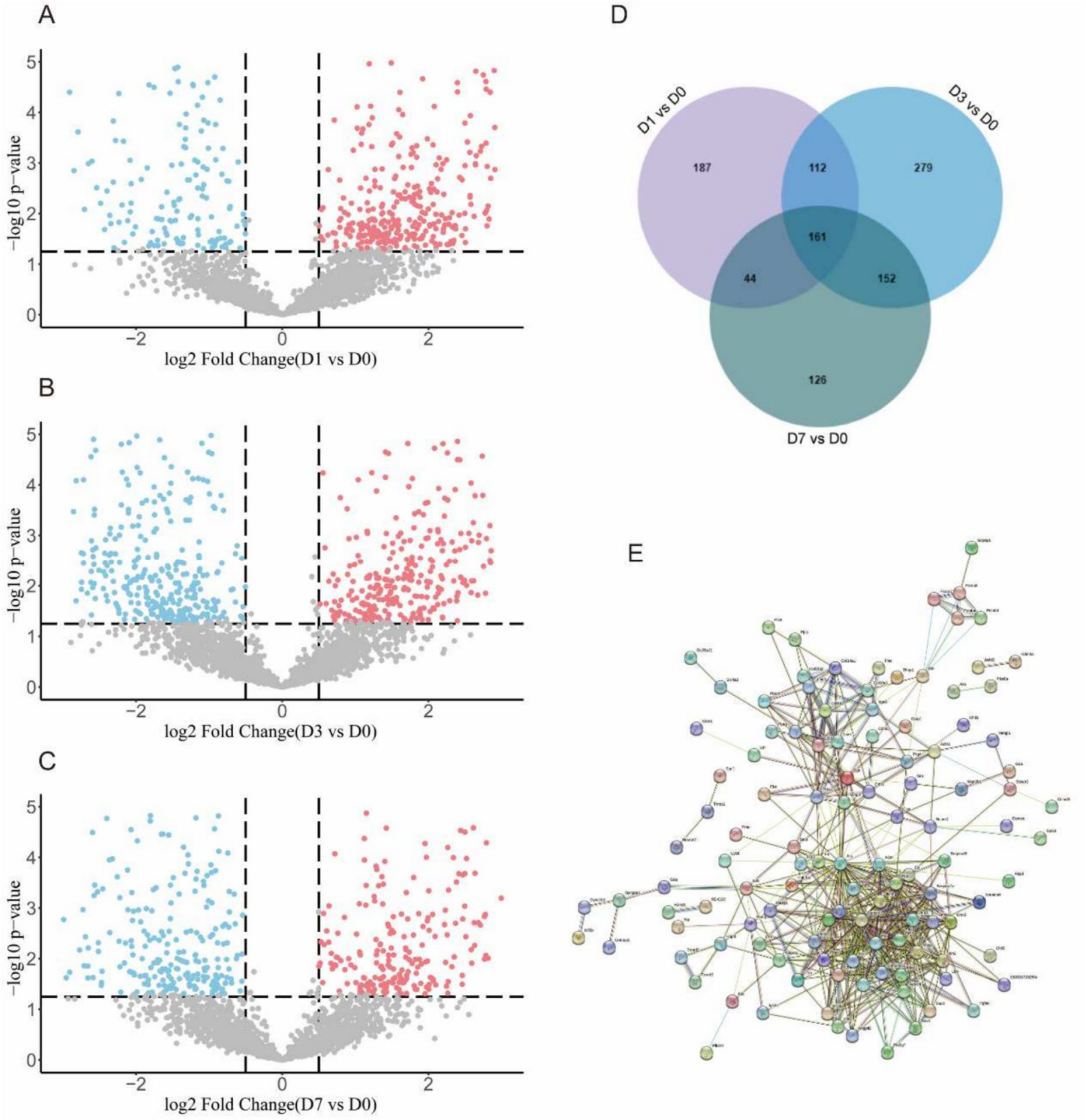
Identification of DEPs in sepsis plasma. **A**–**C** Volcano plot of pairwise comparisons of the plasma proteomes of Day 1, Day 3, and Day 7 after CLP against control. The analyses were performed using the student’s *t*-test (permutation FDR 0.05). Up- and downregulated proteins were highlighted in red and blue, respectively. The plots indicated the gene names of the represented proteins. Light gray data points represent proteins with non-significant *P* values (*P* > 0.05) and/or insignificant fold changes (−0.5 < FC < 0.5). **D** DEPs in septic plasma that alter with time by Venn plot. **E** PPI network illustrating the interactions among proteins that exhibit dynamic changes during sepsis

According to our findings (Fig. 1A–C), MS identified 6,578 plasma proteins in septic young mice. On D1 of sepsis, there were 505 DEPs in the plasma, including 343 proteins that were upregulated and 162 proteins that were downregulated (corresponding to 504 genes). On D3, a total of 706 DEPs were identified, with 337 proteins upregulated and 369 proteins downregulated (corresponding to 704 genes). On D7, 483 DEPs (corresponding to 483 genes) were identified, comprising 244 upregulated proteins and 239 downregulated proteins. Research results indicate that, among the 6578 detected proteins, further analysis of three gene subsets (D1 vs D0, D3 vs D0, D7 vs D0) via Venn diagram revealed that in the plasma of CLP-induced septic mice, 161 proteins exhibited changes (upregulation or downregulation) across D1, D3 and D7 (Fig. 1D), of which adiponectin (Adipoq) exhibited upregulated expression at D1 and D3 time points followed by downregulation at D7. To investigate the relationships among the discovered DEPs, PPI network was created using the STRING database, demonstrating the complicated interactions among many genes (Fig. 1E).

### Characteristics of DEPs

**Fig. 2:**
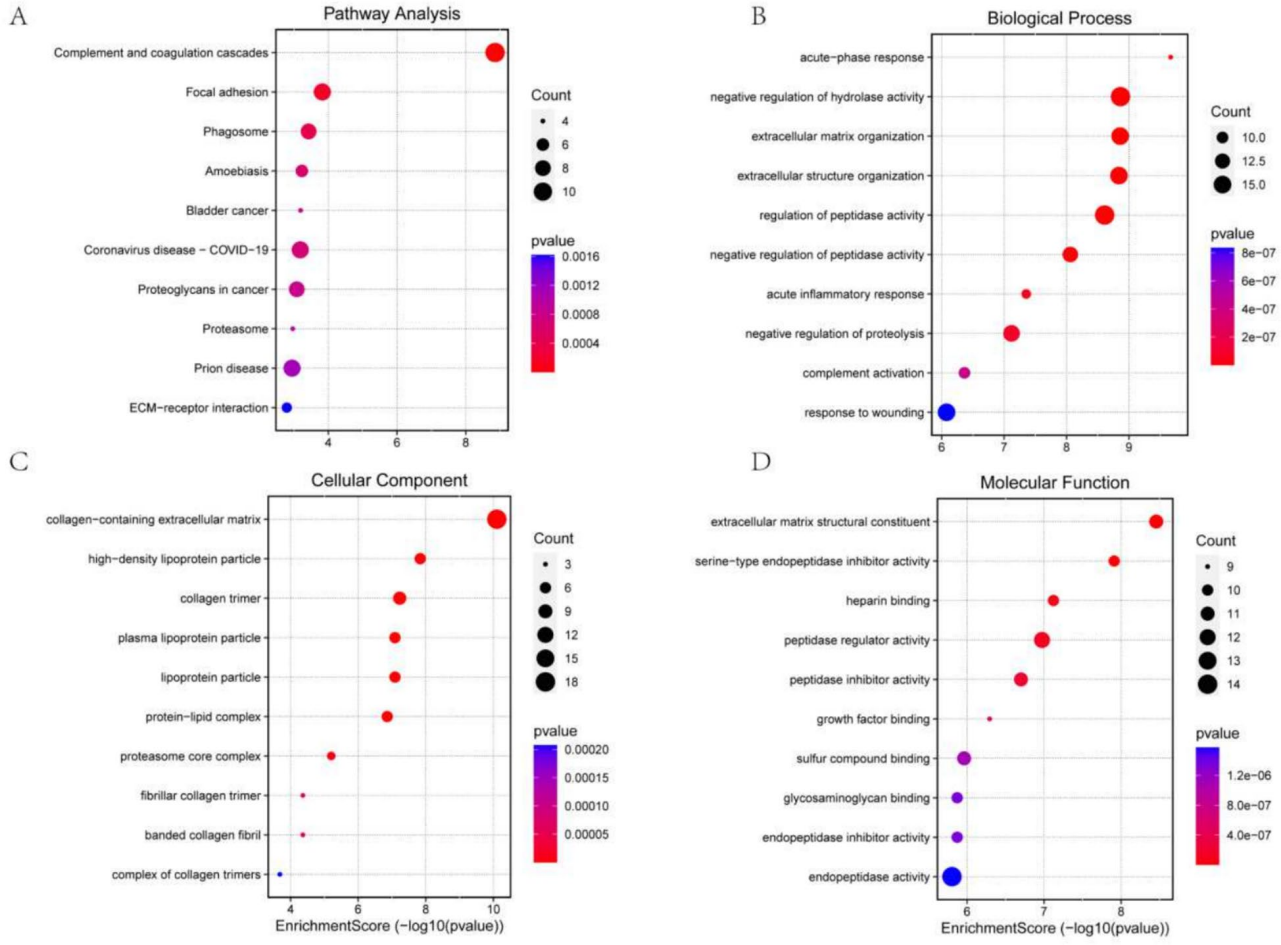

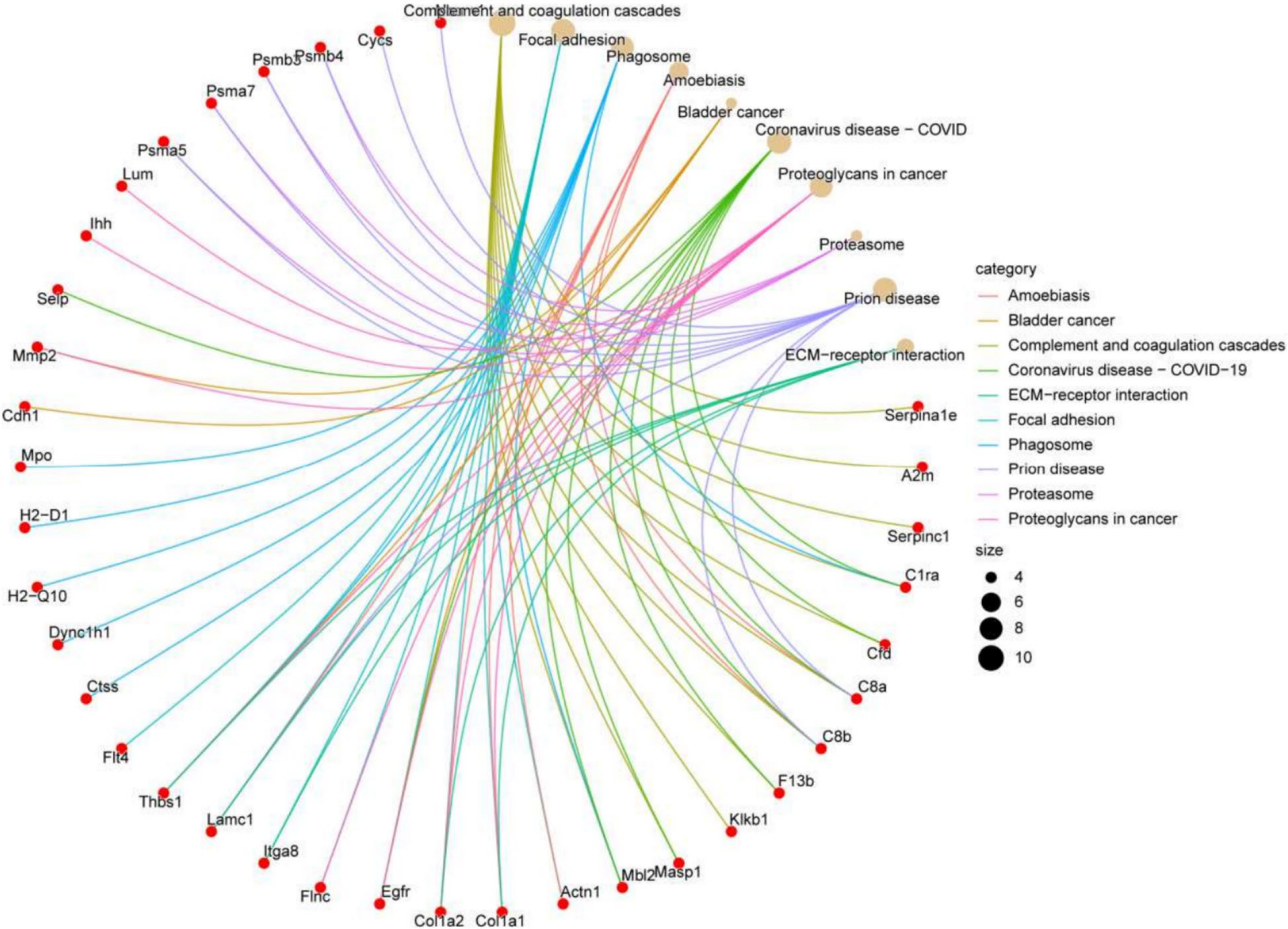
1 Proteomic characterization of DEPs in plasma from septic mice. **A** The top 10 most enriched KEGG signaling pathways. Bioinformatics analysis revealed the top 10 most significantly enriched biological process terms (**B**), cellular component terms (**C**), and molecular function terms (**D**) based on differential protein abundance. The X-axis denotes the percentage of genes associated with the GO term, while the size of the data points represents the number of enriched genes. The color gradient represents the *P* values from the GO and KEGG enrichment analyses, with decreasing *P* values indicated by progressively redder hues. 2 Proteomic characterization of DEPs in plasma from septic mice. The associated proteins from the top 10 most significantly enriched signaling pathways in the KEGG pathway enrichment analysis

In this study, functional enrichment analysis was done to identify the discovered DEPs according to their functions using GO and KEGG analyses. GO is a standardized bioinformatics framework that classifies gene and protein functions into three categories: cellular component, molecular function, and biological process. KEGG is a database that consolidates biological, chemical, and systemic information, emphasizing genes, proteins, and metabolic pathways. KEGG analysis elucidates the metabolic pathways and connections of genes and proteins inside organisms. Through the analysis of these pathways, we can acquire insights about intracellular signaling, metabolic processes, and various biological activities (Fig. 2). The most significantly enriched proteins were connected to complement and coagulation cascades, focal adhesion, and phagosome pathways (Top 3).

### Supervised correlation analysis of proteomic signatures

**Fig. 3:**
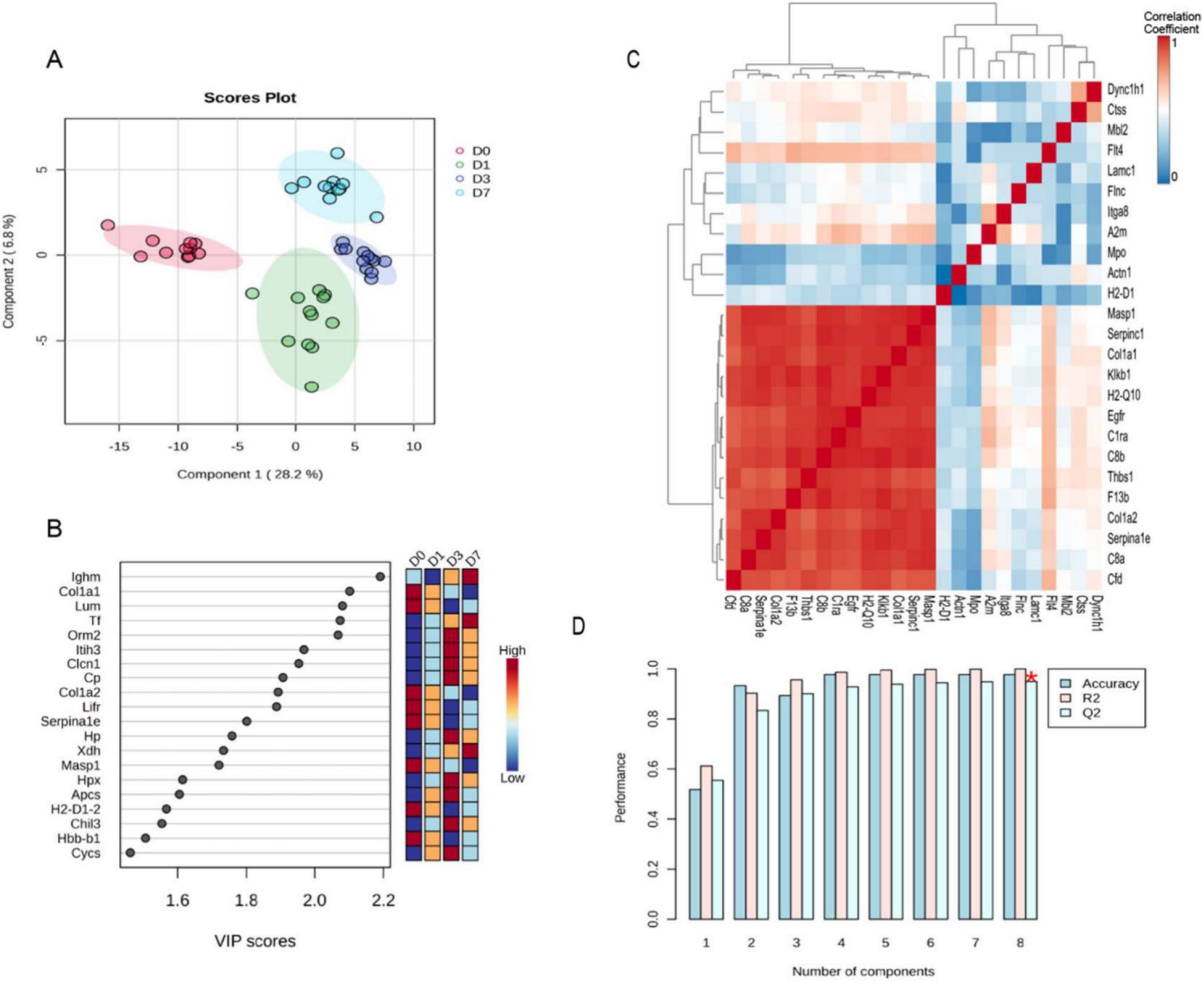
Supervised correlation analysis of proteomic signatures. **A** Partial least squares discriminant analysis (PLS-DA) multidimensional scaling plot of core proteins at different time points. **B** Variable Importance in the Projection (VIP) scores. **C** Correlation analysis, represented as a Pearson correlation coefficient heatmap. **D** Assessment of the predictive capability of the variable model

Proteomic analysis of 161 proteins involved in dynamic alterations was related with sepsis plasma. Partial least squares discriminant analysis (PLS-DA) demonstrated substantial variations in gene expression at several time periods following CLP therapy, with strong intra-group homogeneity (Fig. 3A). Subsequently, we performed variable importance in projection (VIP) ranking on the 161 differentially expressed plasma proteins, with the top 20 proteins depicted in Fig. 3B. Within the VIP scoring analysis, AT III, CFD, Col1α1, EGFR, and Thbs1 registered VIP scores of 1.0, 1.4, 2.0, 0.7, and 1.2, respectively (Supplementary materials). Conventionally, variables with VIP scores exceeding 1.0 are designated as significant discriminatory features and potential biomarker candidates, given their substantial contribution to the model's class-distinguishing capability. Additionally, we selected the top 25 proteins associated with the three most significantly enriched KEGG pathways for correlation analysis (Fig. 3C), showing the Pearson correlation coefficients as a heatmap (red indicating positive correlation and blue indicating negative correlation). Concurrently, we examined the predictive capacity of the model for these 25 proteins; the Q2 value for individual proteins exceeded 0.5 (Fig. 3D), whereas evaluations comprising two or more proteins neared 1. Collectively, our results implied that these 25 proteins demonstrated significant predictive capabilities, with the inclusion of numerous proteins increasing the model's predictive ability.

### Candidate plasma biomarkers

**Fig. 4:**
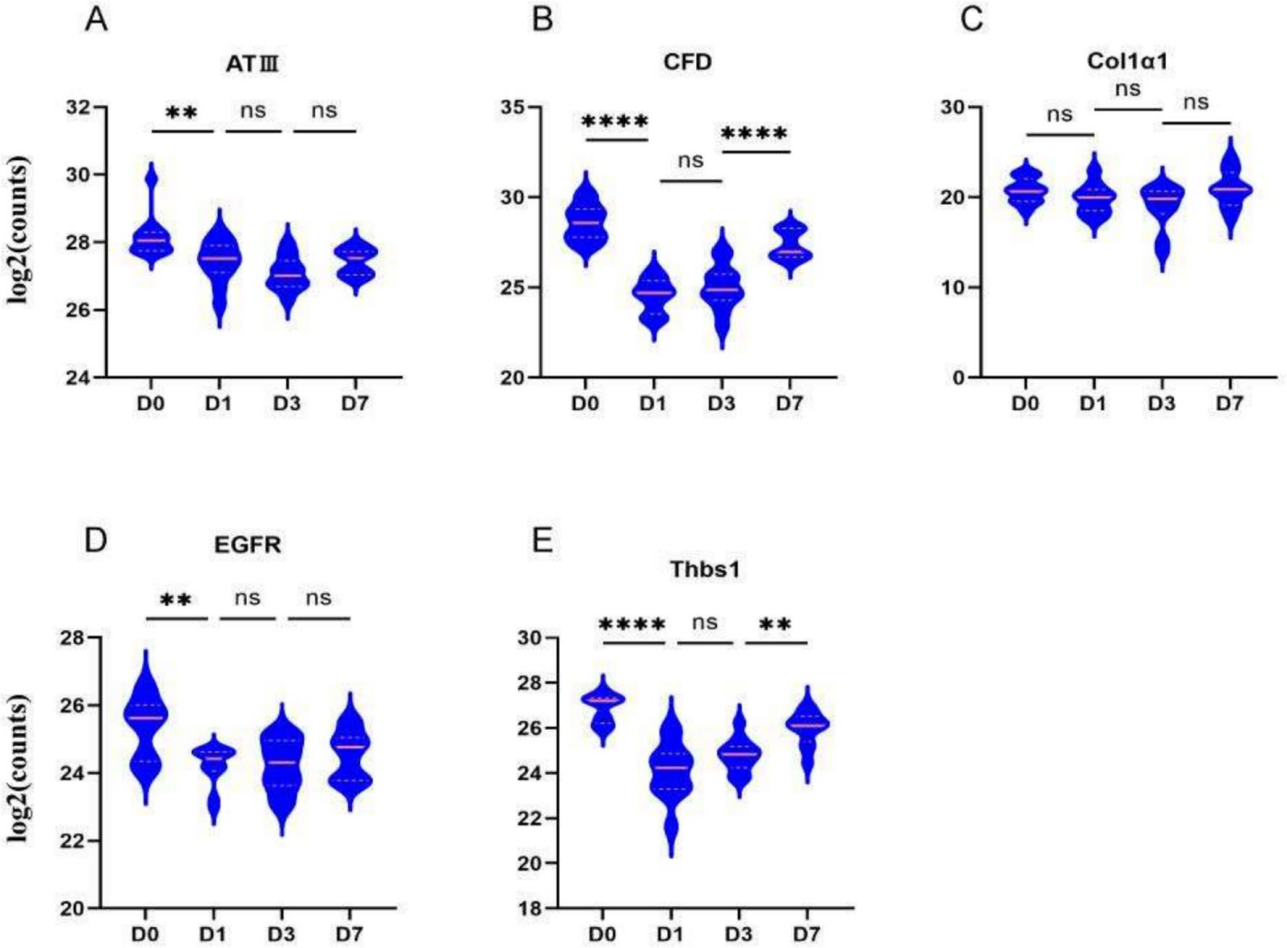
Changes in potential biomarkers at several time points within 1 week in sepsis mice. Violin plot showing the variations in abundance of differentially expressed proteins (AT III, CFD, Col1α1, EGFR, and Thbs1) in septic mice over the course of 1 week. Following CLP-induced sepsis, the plasma abundance of AT III, CFD, EGFR, and Thbs1 was significantly reduced at Day 1, followed by a gradual increase and recovery, whereas Col1α1 did not exhibit this significant trend. *P < 0.05, **P < 0.01, ****P < 0.0001. *ns* non-significant

**Fig. 5:**
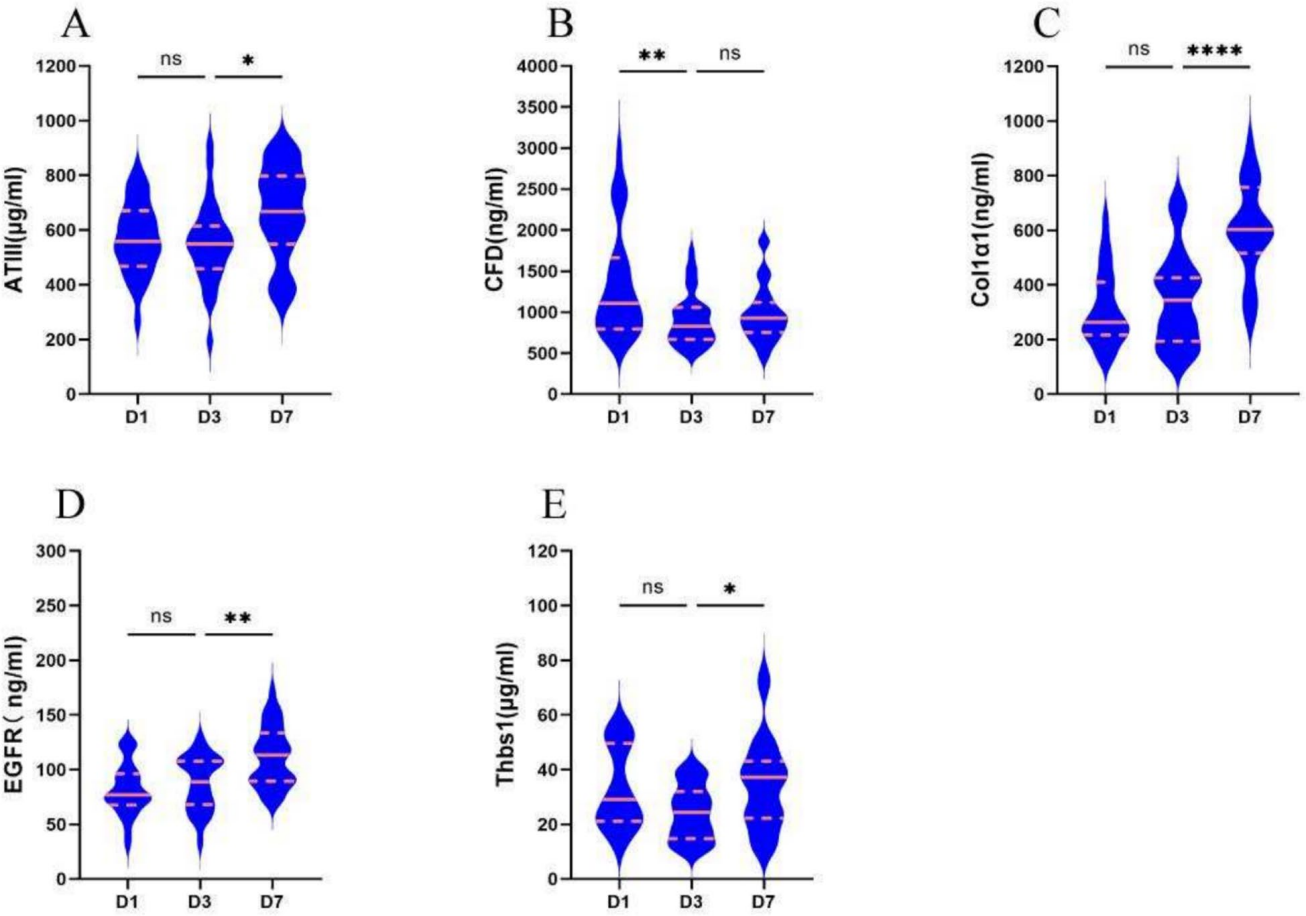
Changes in potential biomarkers (AT III, CFD, Col1α1, EGFR, and Thbs1) at several time points within 1 week in pediatric sepsis. In pediatric patients diagnosed with sepsis on Day 1, plasma levels of AT III, Col1α1, and EGFR were diminished, subsequently increasing progressively, while CFD and Thbs1 remained down on Day 3 and recovered by Day 7. *P < 0.05, **P < 0.01, ****P < 0.0001. *ns* non-significant

The characterization of the plasma proteome demonstrating significant differential expression during sepsis (Fig. 2E) principally comprises three major types of proteins: (1) Proteases involved in the complement and coagulation cascades, including serpina1e, A2m, AT III, C1ra, CFD, C8a, C8b, F13b, Klkb1, Masp1, and Mbl2; (2) Extracellular proteins participating in focal adhesion processes, such as Actn1, Col1α1, Col1α2, EGFR, Flnc, Tga8, Lamc1, Thbs1, and Flt4; and (3) Proteins associated with phagosome activity, including Actn1, Col1α1, Col1α2, C8a, C8b, and Lamc1. A total of 161 differentially expressed proteins were input into the STRING platform to screen for mechanism-anchored biomarkers through protein–protein interaction (PPI) network analysis. Integrated KEGG pathway clustering revealed co-regulated modules in the complement system–coagulation cascade and focal adhesion pathways. Concurrently, GO-BP enrichment analysis localized key biological processes, identifying significant accumulation of differentially expressed proteins within the acute-phase response. Protein selection was guided by bioinformatics analyses—including functional protein association networks (STRING), KEGG pathway enrichment, GO annotation, and PLS-DA—to identify biologically relevant processes. From the two most significantly enriched pathways, secretory proteins compatible with commercially available assay kits were systematically selected as candidate biomarkers (AT III, CFD, Col1α1, EGFR, and Thbs1). Final validation was performed via ELISA in a prospectively collected patient cohort. We calculated the abundance of all proteins in each sample (log2 transformed) and found that the candidate plasma biomarkers demonstrated varying degrees of decrease following the onset of sepsis, with trends in both the validation cohort and training cohort showing remarkable similarity (Table 1, Figs. 4 and 5). Following CLP-induced sepsis, the plasma abundance of AT III, CFD, EGFR, and Thbs1 was significantly reduced at D1, followed by a gradual increase and recovery, whereas Col1α1 did not exhibit this significant trend. In pediatric patients diagnosed with sepsis on D1, plasma levels of AT III, Col1α1, and EGFR were diminished, subsequently increasing progressively, while CFD and Thbs1 remained down on D3 and recovered by D7.

**Table 1:** Longitudinal changes in plasma biomarker concentrations among 66 sepsis children

Protein	Time (day)	Concentration (mean ± SD)	*P* value
AT III (μg/mL)	D1	570.48 ± 25.61	–
	D3	551.69 ± 29.38	0.8945
	D7	665.65 ± 32.41	0.0198
Col1α1 (ng/mL)	D1	313.01 ± 29.07	–
	D3	351.94 ± 35.71	0.6948
	D7	599.17 ± 37.07	< 0.0001
CFD (ng/mL)	D1	1360.14 ± 134.25	–
	D3	913.19 ± 59.74	0.0019
	D7	981.61 ± 64.62	0.8376
EGFR(ng/mL)	D1	81.54 ± 4.54	–
	D3	87.56 ± 5.29	0.6861
	D7	111.43 ± 5.04	0.0036
Thbs1(μg/mL)	D1	32.87 ± 3.26	–
	D3	24.31 ± 2.15	0.1296
	D7	34.76 ± 3.52	0.0441

The *P* values relate to the statistical analysis results for Day 3 vs. Day 1 and Day 7 vs. Day 3

### Characteristics of the study population

This study enrolled a total of 66 pediatric subjects, with demographic and clinical characteristics of the patients described in Table 2. The average age of the participants was 42.4 months, with males forming 63.6%, and the mean APACHE II (D1) score was 12.68. All patients received guideline-directed therapeutic and nursing interventions per standard protocols. As presented in Table 2, respiratory tract infections predominated among enrolled subjects (71.2%), followed by central nervous system infections (18.2%), with the remainder comprising genitourinary, gastrointestinal, and other infections (10.6%). Pharmacological interventions included antimicrobial therapy (95.5% of patients), glucocorticoid administration (51.5%), and intravenous immunoglobulin (57%). Vasoactive agents were required in 27.2% of cases, while respiratory support involved non-invasive ventilation (43.9%) and mechanical ventilation (9.1%). Crucially, all study participants survived through the observation period with no fatalities recorded. Laboratory testing revealed that the white blood cell (WBC) count was (11.12 ± 0.94) × 10⁹/L on D1, decreasing by D3 (*P* value of 0.0107). The percentage of neutrophils was highest on D1, subsequently dropping (P values of 0.0005 and 0.0445, respectively). Platelets revealed abnormal biphasic alterations, initially dropping at the onset of sepsis, followed by a recovery in platelet count and thrombocytosis. C-reactive protein (CRP) and procalcitonin (PCT) gradually normalized over the course of the disease, with no significant statistical differences found between time periods. Immune function indices, including lymphocyte subsets and immunoglobulins (A, G, M), were lower than age-specific reference values.

**Table 2:** Demographic characteristics of the pediatric sepsis cohort

Characteristics		Total patients (*N* = 66) (mean ± SD)	*P* value
Male, *n* (%)		42 (63.6)	–
Age, months		42.4	–
APACHE II of admission		12.68	–
Location of infection			
Respiratory system, *n* (%)		47 (71.2)	–
Neurological system,		12 (18.2)	–
Others		7 (10.6)	–
Treatment at PICU			
Antimicrobial, *n* (%)		63 (95.5)	–
Vasoactive, *n* (%)		18 (27.2)	–
Glucocorticoid, *n* (%)		44 (51.5)	–
IVIG, *n* (%)		35 (57)	–
MV, *n* (%)		6 (9.1)	–
NIV, *n* (%)		29 (43.9)	–
Outcome			
Survivors, *n* (%)		66 (100)	–
Non-survivors, *n* (%)		0 (0)	–
Laboratory findings			
WBC (10^**9**^/L)	D1	11.12 ± 0.94	–
	D3	8.16 ± 0.52	0.0107
	D7	8.62 ± 0.52	0.9128
N (%)	D1	63.31 ± 2.68	–
	D3	48.72 ± 2.79	0.0005
	D7	38.57 ± 2.97	0.0445
PLT (10^**9**^/L)	D1	293.42 ± 17.63	–
	D3	330.56 ± 19.47	0.4338
	D7	434.23 ± 32.61	0.0063
CRP (mg/L)	D1	31.66 ± 5.63	–
	D3	19.97 ± 6.44	0.2472
	D7	2.29 ± 0.5	0.0741
PCT (ng/ml)	D1	3.59 ± 1.41	–
	D3	1.38 ± 0.38	0.5791
	D7	0.22 ± 0.08	0.9078
PT (s)		11.04 ± 0.13	–
APTT (s)		30.34 ± 0.96	–
INR		0.97 ± 0.01	–
D-Dimer (mg/L)		1.49 ± 0.33	–
Fbg (g/L)		4.03 ± 0.38	–
IgA (g/L)		0.78 ± 0.09	–
IgG (g/L)		8.16 ± 0.41	–
IgM (g/L)		0.97 ± 0.06	–
C3 (g/L)		1.08 ± 0.04	–
C4 (g/L)		0.44 ± 0.13	–
CD3 + (%)		55.18 ± 2.03	–
CD4 + (%)		30.48 ± 1.58	–
CD8 + (%)		20.3 ± 1.15	–
CD19 + (%)		30.3 ± 2.14	–
CD16 + CD56 + (%)		12.16 ± 1.7	–
CD3 + CD4 + CD8 + (%)		0.75 ± 0.12	–
CD4 +/CD8 + T (%)		1.7 ± 0.14	–
CD3 + CD4-CD8- (%)		8.65 ± 0.79	–
IL-2 (pg/ml)		15.58 ± 14.84	–
IL-4 (pg/ml)		2.24 ± 0.42	–
IL-6 (pg/ml)		307.53 ± 182.19	–
IL-10 (pg/ml)		39.57 ± 33.18	–
TNF-α (pg/ml)		30.1 ± 5.74	–
INF-γ (pg/ml)		90.24 ± 39.98	–

*Fbg* Fibrinogen, *INR* International Normalized Ratio, *PT* Prothrombin Time, *APTT* Activated Partial Thromboplastin Time, *IgA* Immunoglobulin A, *IgG* Immunoglobulin G, *IgM* Immunoglobulin M, *C3* Complement 3, *C4* Complement 4, *WBC* White Blood Cell, *N* Neutrophil, *PLT* Platelet, *MV* Mechanical ventilation, *NIV* Non-invasive ventilation, *IVIG* intravenous immunoglobulin. The *P* values relate to the statistical analysis results for Day 3 vs. Day 1 and Day 7 vs. Day 3

### Correlation analysis of candidate biomarkers

**Fig. 6:**
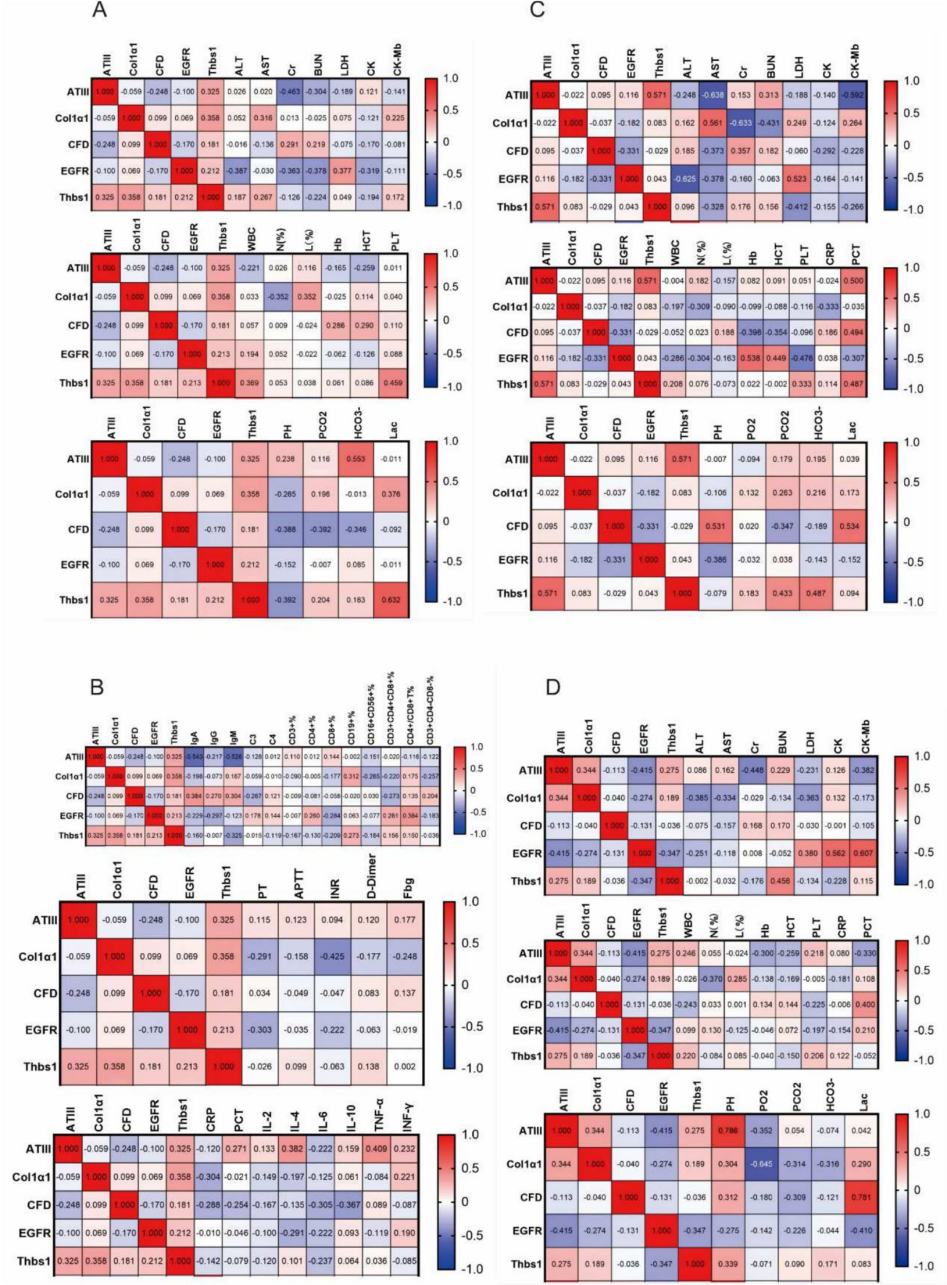
Correlation coefficients between potential biomarkers and clinical laboratory indicators. **A**, **B** correlation coefficients of Day 1. **C** correlation coefficients of Day 3. **D** correlation coefficients of Day 7

A correlation analysis (using Pearson correlation coefficients) was undertaken between the five screened putative biomarkers and common laboratory indices. The five putative sepsis plasma biomarkers (AT III, CFD, Col1α1, EGFR, Thbs1) revealed certain relationships with clinical laboratory indices related to hematology, biochemistry, blood gas analysis, coagulation function, and immune function (Fig. 6). Enrichment pathways demonstrated considerable enrichment of proteins linked with coagulation and complement. Therefore, we focused our analysis on associations between coagulation function and immunological function. The results showed that AT III was negatively linked with IgA, IgG, IgM, and C3, with Pearson indices R of −0.543, −0.217, −0.526, and −0.128, respectively. CFD was positively connected with IgA, IgG, and IgM, with correlation indices of 0.384, 0.270, and 0.304, respectively, and negatively correlated with C3 (−0.267). Col1α1, CFD, EGFR, and Thbs1 were negatively connected with inhibitory CD8 + cells (−0.177, −0.058, −0.284, and −0.209, respectively), while Col1α1, EGFR, and Thbs1 were favorably correlated with B cells (CD19 +) (0.312, 0.063, and 0.273, respectively). Col1α1, CFD, EGFR, and Thbs1 were favorably linked with the CD4 +/CD8 + ratio (0.175, 0.135, 0.384, and 0.150, respectively). AT III was favorably connected with PT, APTT, INR, D-Dimer, and Fbg, while Col1α1 and EGFR were negatively correlated with PT, APTT, INR, D-Dimer, and Fbg. CFD was positively connected with Fbg (0.137), and Thbs1 was positively correlated with D-Dimer (0.138).

## Discussion

Sepsis can cause dynamic changes in proteomics and metabolomics within a short time. Metabolites, as final products of gene and protein functions, can directly reflect the real-time physiological and pathological states of an organism, such as energy metabolism disorders and oxidative stress in sepsis. They provide dynamic information closest to the phenotype [13] and capture early pathophysiological disturbances in diseases, like energy metabolism collapse and oxidative stress in sepsis [14]. But the proteome reflects the functional expression of the genome, and proteomics can reveal upstream regulatory mechanisms, such as inflammatory pathway proteins and coagulation factors, and explain the drivers of metabolic changes. Therefore, proteomics is now regarded as the most suitable tool for understanding gene functions [15]. As spatial proteomics and high-throughput proteomics technology have evolved, proteomics can now quantify hundreds of proteins and their abundances to unravel probable molecular pathways behind pediatric sepsis and to identify tailored biomarkers and therapy methods [16]. Our MS results consistent with prior studies [10], demonstrating that the plasma proteome involved with coagulation and complement undergoes large abundance changes during sepsis. Indeed, the complement system, as a quick and powerful immune surveillance mechanism, exerts enormous influence on both healthy and changed host cells, as well as invading pathogens. Upon recognition of pathogen-associated molecular patterns (PAMPs) during microbial invasion, the host triggers one or more complement activation pathways aimed at eradicating the microbial intruders. Furthermore, during infection, the interplay between the complement and coagulation systems increases local coagulation and limits the spread of microorganisms via the circulation [17].

AT III is the primary inhibitor of the coagulation cascade, synthesized by the liver as a glycoprotein that irreversibly inhibits serine proteases (Xa and IIa) in a 1:1 ratio, forming a protease-AT II complex that is needed for anticoagulation [18]. Beyond its anticoagulant effects, AT III affects the immune system and may alter endothelial cell activity [19]. Increasing data show that AT III possesses anti-inflammatory [20–22] and renoprotective effects [23–26]. Recent research revealed that AT III could inhibit the serine protease TMPRSS2, thereby decreasing viral load in COVID-19 patients and early SARS-CoV-2 infection [27]. AT III is a marker of DIC in sepsis patients and is significant for prognosis [28, 29], and levels below 20% being associated with fatal results [30]. Overall, our data supported the hypothesis that AT III may serve more as an inflammatory response regulator than a coagulation inhibitor. Inthorn et al. [31] observed that AT III supplementation greatly improved respiratory, hepatic, and renal failure while also reducing certain inflammatory markers. Our findings demonstrated that AT III in plasma dropped and then progressively returned in the detection of pediatric sepsis, while the association between AT III expression levels in plasma and sepsis prognosis need additional validation through investigation.

The complement system is a proteolytic cascade that can be activated by the classical, lectin, and alternative pathways. CFD amplifies all complement-mediated bactericidal actions and plays a vital part in the membrane assault complex directed against bacteria, mediated by mannose-binding lectin [32–34]. Furthermore, CFD in the gut has a critical function in the clearance of Escherichia coli [35]. Research has demonstrated that CFD showed a negative correlation with the pro-inflammatory factor IL-6 and a positive correlation with the anti-inflammatory factor IL-4 [36]. This indicated that CFD could serve as an anti-inflammatory agent. Our data revealed that in the context of sepsis, CFD levels decline to a minimum on D3 and rebound by D7. Nevertheless, additional trials are required to elucidate the precise processes and signaling pathways that underlie its anti-inflammatory properties.

Col1α1, the primary constituent of type I collagen, is broadly distributed in parenchymal organs and interstitial connective tissues across the body [37]. Col1α1 is crucial for controlling intercellular adhesion and differentiation, as well as fortifying diverse tissues in the body [38]. Col1α1 controls cell proliferation, metastasis, invasion, and angiogenesis [39, 40]. Recent research on the molecular mechanisms of sepsis-induced diaphragm dysfunction demonstrated that the Col1α1 gene, connected to cellular adhesion, is downregulated 24 h after lipopolysaccharide (LPS) injection [41]. Col1α1 mutations in animal models of osteogenesis imperfecta led to reduced diaphragm mass and contractility [42]. Our study demonstrated reduced plasma Col1α1 expression levels after sepsis, indicating a probable relationship with pediatric sepsis-induced lung injury.

The ErbB family contains the archetypal members, EGFR/ErbB1, ErbB2, ErbB3, and ErbB4, which are cell surface growth factor receptors found in numerous developing mammalian organs. Previous experiments have showed that overexpressing EGFR and ErbB4 protects mice from acute pancreatitis [43]. In vitro and in vivo investigations revealed that EGFR is intricately connected to the maintenance and repair of normal epithelial cells [44]. ErbB1 inactivation induces hemorrhagic enteritis, which is analogous to necrotizing enterocolitis [45]. Furthermore, previous studies have demonstrated that NRG1-ErbBs signaling has cytoprotective, anti-inflammatory, and anti-remodeling effects [46–48]. In colonic epithelial cells, EGFR activation promotes proliferation [49], lowers cytokine-induced apoptosis [50], and speeds migration/wound repair [51, 52]. Increasing data suggested that ErbB signaling deficits occur under a range of intestinal inflammatory settings [53–55]. Our data demonstrated that EGFR is expressed at low levels in plasma during the early stages of sepsis; however, its likely involvement in these illnesses is unknown, and it may be implicated in the endothelial response to infection/inflammation.

Thbs1 modulates TGF-β activation, impacting wound healing, proliferation, differentiation, and cytokine responses [56]. Thbs1 has been found as a component of a risk categorization model for pediatric septic shock that combines multiple biomarkers [57]. A single-center cohort study suggested [58] that Thbs1 is a possible prognostic marker for poor outcomes in neonatal patients with pneumonia sepsis. In human cells, Thbs1 suppresses IL-1β and caspase-1 mRNA, not LPS-induced NLRP3 [59]. Previous research has demonstrated that Thbs1 has context-specific pro- and anti-inflammatory actions [60–62], with Thbs1 produced in response to inflammation, supporting the resolution of inflammatory processes and facilitating phagocytosis of wounded cells [63, 64]. During the early phases of injury and inflammation, high levels of Thbs1 promote dendritic cell tolerogenicity to antigens, therefore halting the inflammatory response. Thbs1 regulates the creation and activation of pro-inflammatory cytokine IL-1β in human and murine macrophages. Thbs1 suppresses IL-1β mRNA induction in an NF-κB/AP-1-dependent mechanism [59]. Thbs1 levels suppress the production of LPS-induced IL-1β mRNA and protein in human macrophages. Our findings revealed that Thbs1 is expressed at low levels in the plasma of children with sepsis, making it a viable biomarker for sepsis in this population. As a result, the biological function of Thbs1 during sepsis deserves deeper research.

Coagulation activation during infection may be helpful [65, 66], as it increases platelet activation through the production of thrombin, enhancing the creation of microthrombi in inflammatory and infectious responses. This condition, known as immunothrombosis, tries to entrap invading microorganisms and restrict their dissemination. Research results revealed [67] that thrombocytopenia at the time of admission was associated with higher mortality and deregulation of host responses during sepsis. Notably, the kinetics of platelets during sepsis generally exhibit a biphasic pattern, defined by an initial fall within the first few days (1–4 days), followed by a subsequent increase in platelet counts [68]. Our findings revealed that platelet counts began to rise after Day 3.

Lymphocytopenia induced by sepsis is a transient event, with lymphocyte numbers eventually returning to pre-sepsis levels. A growing body of evidence supported that the immune status of patients is related to the ultimate outcome of sepsis. CD4 + T lymphocytes can activate B cells and effector T cells through the release of various factors, thereby upregulating immune function, while CD8 + T lymphocytes possess surveillance and cytotoxic functions. Studies have found [69, 70] that a decreased CD4 +/CD8 + ratio is closely associated with immunosuppression and poor prognosis. In response to infectious agents, B cells not only secrete IgM and IgG but also phagocytize, process, and present antigens to T cells to generate humoral immunity [71, 72]. Previous studies have reported [73] an increase in B cell numbers in whole blood of sepsis patients, whereas most other studies show a decrease in B cell numbers in severe sepsis patients [74–77]. Additionally, septic shock patients often present with hypogammaglobulinemia [78], and levels of IgG, IgM, and IgA at the time of diagnosis are directly associated with survival rates [79]. Our findings indicated that, at the time of sepsis identification, lymphocyte subsets and gamma globulin levels were lower than normal reference values, which may reflect impaired immune function during sepsis.

### Limitations

Our study also highlighted a few limitations. Firstly, the validation cohort of this work utilized plasma from experimental animals with a disease model to screen for potential markers. Although these experimental animals share a comparable genetic background with people, and characteristics such as body weight and age can be easily controlled to strengthen the comparability of the trials, they do not fully reflect the heterogeneity of the human situation. Secondly, when acquiring clinical laboratory parameters, some data (e.g., immune cells, cytokines, etc.) lacked information from the intermediate and late stages of sepsis (D3 and D7), which hindered a thorough display of their dynamic changes during sepsis. Third, this study constitutes a single-center investigation. A promising future direction holds the potential to refine clinically relevant sepsis biomarkers through the integration of multi-omics approaches combined with longitudinal patient follow-up, leveraging machine learning pipelines to enhance generalizability to broader populations. In subsequent investigations, we will expand the validation cohort to further explore biomarker-driven clinical applications, such as diagnostic cutoff values and predictive modeling.

## Conclusion

In this proteomic examination of plasma in septic young mice, we detected 161 significantly increased proteins at distinct time intervals throughout the first week of sepsis (D1, D3, D7). According to KEGG pathway enrichment analysis, the complement and coagulation cascades were most closely correlated to the differentially expressed proteins, followed by focal adhesion and phagocyte-related proteins. All five possible plasma indicators declined to varied degrees at the time of sepsis diagnosis and were linked with coagulation and immunological activity. Our exploratory proteomic profile delineates targetable candidates for advancing disease surveillance systems and prognostic modeling frameworks. Furthermore, the signaling pathway mapping establishes referential vectors for mechanistic investigations into host–pathobiont interplay and therapeutic perturbation responses. As a result, more validation studies are needed to establish the causal link between sepsis and increased or decreased expression of any newly found proteins.

## Supplementary Information

Supplementary material 1.

## Data Availability

Data is provided within the manuscript.
