# Supplementary material for: Dynamic alterations and clinical implications of the plasma proteome in pediatric sepsis
Source: Eur J Med Res. 2025 Jul 23;30:659. doi: 10.1186/s40001-025-02933-5 (PMC12285057; doi:10.1186/s40001-025-02933-5)
Supplement: Supplementary file 1 — Supplementary material 1. [file 40001_2025_2933_MOESM1_ESM.docx]

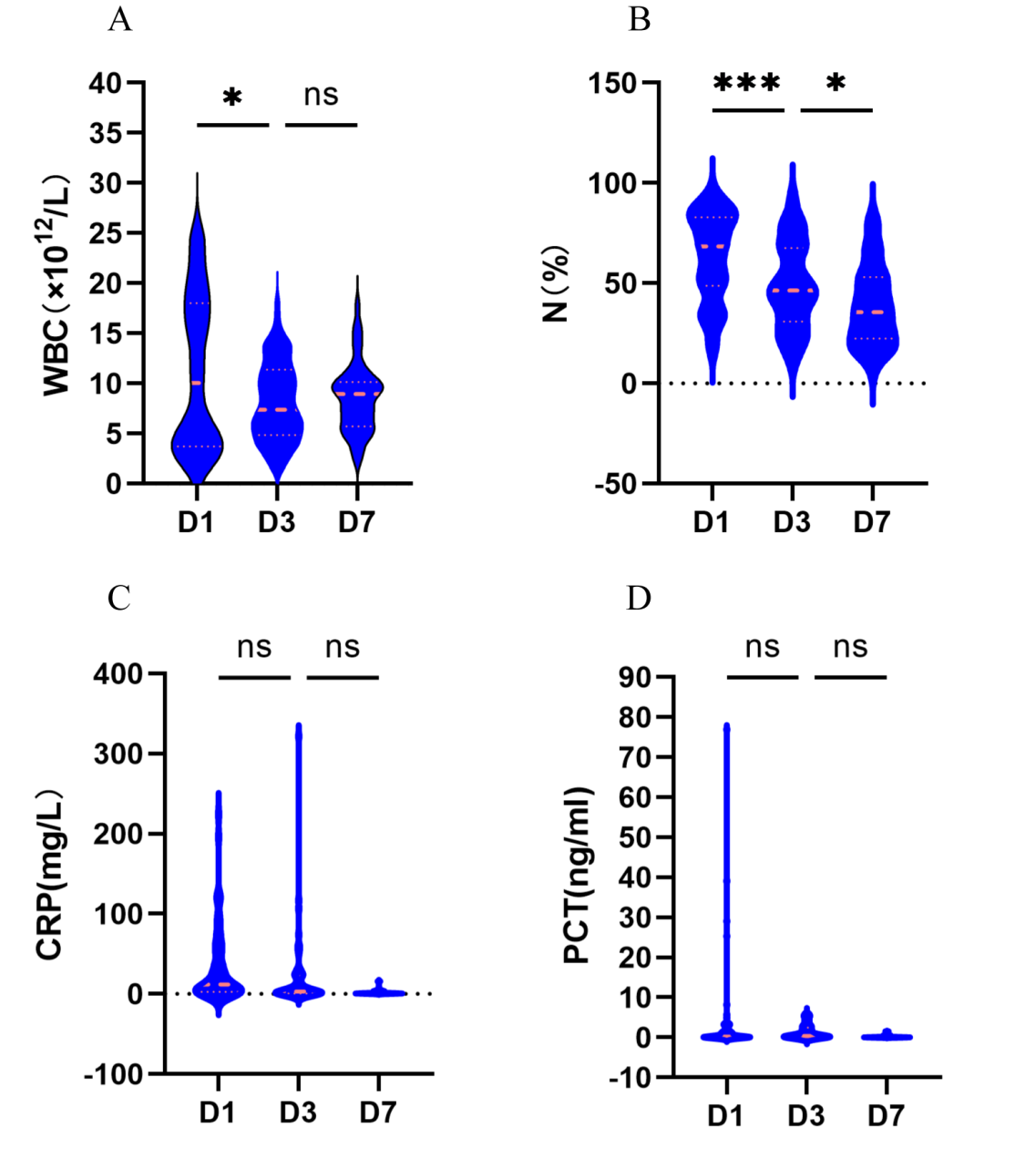


Supplementary Fig.1 Longitudinal changes in laboratory parameters for the sepsis cohort.

A. White blood cell count (WBC). B. Neutrophil percentage. C. C-reactive protein (CRP). D. Procalcitonin (PCT). * P < 0.05, *** P < 0.001. ns, non-significant.
